# MiR-126 promotes esophageal squamous cell carcinoma via inhibition of apoptosis and autophagy

**DOI:** 10.18632/aging.103379

**Published:** 2020-06-18

**Authors:** Mingli Li, Xiangli Meng, Mingxuan Li

**Affiliations:** 1 Department of Life Science and Engineering, Jining University, Qufu, Shandong, China; 2 Department of Nursing, Affiliated Hospital of Jining Medical University, Jining, Shandong, China

**Keywords:** miR-126, esophageal squamous cell carcinoma, STAT3, apoptosis, autophagy

## Abstract

MiRNA-126 (miR-126) has been shown to be involved in various malignancies as well as other biological processes. However, currently, its role in esophageal squamous cell carcinoma (ESCC) is not well understood. The present study is focused on the mechanisms that underlie the effect of miR-126 on cell survival and death (apoptosis and autophagy) in ESCC cells. MiR-126 expression was found to be enhanced in ESCC cells and tissues. Downregulation of miR-126 suppressed cell survival, and TUNEL staining indicated that miR-126 inhibition promoted ESCC cell death. In addition, the production of LC3B and p62 proteins, two autophagy signals, was reduced following miR-126 inhibition. A dual luciferase reporter assay demonstrated that the STAT3 3’-UTR is a direct target of miR-126. Furthermore, *STAT3* knock-down rescued the effects on autophagy and apoptosis caused by miR-126 inhibition in ESCC cells. The results of this study may provide some insight into the molecular and biological mechanisms underlying ESCC generation and contribute to the development of novel therapeutic approaches for ESCC.

## INTRODUCTION

Esophageal cancer (EC) is a fatal disorder typified by a poor clinical outcome owing, in part, to the fact that many patients with EC receive their diagnosis during the late stages of the illness [1, 2]. Although remarkable progress has been made in diagnostic methods and adjuvant chemotherapy, the five-year survival rate of patients with EC remains below 30% [3]. Previous studies have indicated that complex epigenetic and genetic changes may result in the progression of esophageal squamous cell carcinoma (ESCC) [4, 5]. However, a proper understanding of the mechanisms underlying such changes is currently lacking.

MicroRNAs (miRNAs) are a series of conserved small RNAs comprising 20–22 nucleotides; they modulate gene expression by binding to the 3'-UTR of the target mRNA, leading to mRNA degeneration and translational suppression [6]. Current evidence suggests that miRNAs may critically influence various biological processes, including cell death. For example, miR-34a is suppressed in malignancies as well as in multiple cellular processes such as cell proliferation, differentiation, and death [7–9].

MiR-126 has been shown to modulate metastasis as well as proliferation in multiple malignancies via the inhibition of gene expression. For example, miR-126 promotes sensitivity to drugs that counteract malignancy in non-small-cell lung cancer (NSCLC) by targeting vascular endothelial growth factor (VEGF) A [10]. MiR-126 represses proliferation and triggers cell death in hepatocellular carcinoma (HCC), at least in part, by acting on Sox2 [11]. The expression of miR-126 is often significantly suppressed in tissues from patients with gastric cancer. Excessive miR-126 expression inhibits the invasion of malignant cells instead of affecting proliferation *in vitro*. Furthermore, excessive miR-126 noticeably reduces the concentrations of Crk protein, which has been recognized as a direct target of miR-126. Crk knock-down (KD) caused a marked inhibition of GC cell invasion [12]. miRNA expression has been reported in ESCC [13, 14], and specific miRNAs, such as miR-21 and miR-129-2, may participate in ESCC generation [15, 16].

The aim of this study was to explore the impact of miR-126 on ESCC etiology and development. MiR-126 expression was found to be increased in specimens from patients with ESCC as well as in ESCC cell lines. Furthermore, miR-126 silencing impaired ESCC cell proliferation and ESCC generation both *in vivo* and *in vitro*. In addition, our results suggest that miR-126 may participate in ESCC development by suppressing cell death via the downregulation of STAT3.

## RESULTS

### MiR-126 was upregulated in ESCC tissues and cells

**Figure 1:**
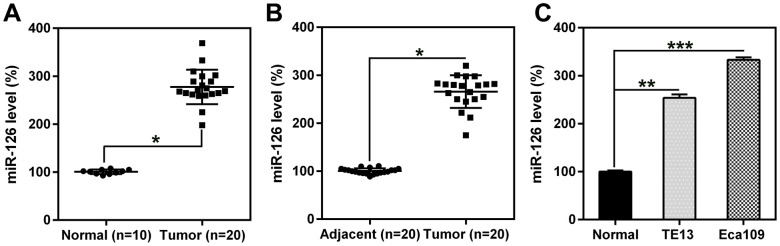
**miR-126 levels in esophageal tissues from patients with ESCC and in ESCC cell lines.** (**A**, **B**) miR-126 expression in ESCC (n = 20) and healthy (n = 10) specimens, compared to that in adjacent healthy pancreatic tissues (n = 20). (**C**) miR-126 expression in TE13 and Eca109 cell lines and healthy esophageal cells. Results are displayed as the average ± SD. *P < 0.05, **P < 0.01, and ***P < 0.001.

miR-126 expression in ESCC (n = 20) and esophageal specimens from healthy volunteers (n = 10) was investigated using qPCR. Expression levels in the ESCC malignant group were considerably higher than those in the normal group (Figure 1A). Furthermore, miR-126 expression in 20 human ESCC and 20 adjacent normal tissue specimens was also evaluated. The results showed that miR-126 expression was enriched in the human ESCC specimens, indicating a higher level of miR-126 in ESCC tumors than in the surrounding normal esophageal tissues (Figure 1B). In addition, miR-126 expression was higher in the two ESCC cell lines than in normal esophageal cells (Figure 1C). The above findings indicate that ESCC tissues and cells express high levels of miR-126.

### MiR-126 silencing inhibited ESCC tumorigenesis *in vivo*

**Figure 2:**
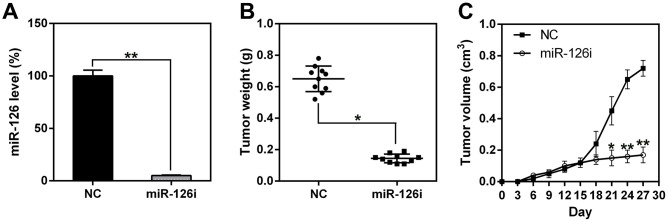
**miR-126 silencing suppresses xenograft-induced ESCC tumorigenesis.** (**A**) miR-126 expression in esophageal tissues was measured using qPCR. (**B**) TE13 cells treated with shRNA-miR-126 or NC were injected subcutaneously into nude BALB/c mice. Mice were sacrificed at day 21 after inoculation, and the tumors were removed and weighed. (**C**) Tumor growth curve over 27 d. Results are displayed as the average ± SD. *P < 0.05, **P < 0.01, and ***P < 0.001.

To assess the role of miR-126 in xenograft ESCC generation, we subcutaneously injected nude BALB/c mice with TE13-miR-126i or TE13-NC cells and observed the animals every day for tumor formation. First, miR-126 expression was measured in esophageal tissues to confirm miR-126 knock-down in the miR-126-silenced mice (Figure 2A). Mice were sacrificed 21 days after the injection, and their esophageal tissues were removed and weighed. The results indicate that the average weight and volume of the miR-126-silenced tumors were much lower than those of the control group tumors, and the average neoplasm weight was also significantly less (Figure 2B, 2C).

### MiR-126 silencing reduced cell viability and induced cell death in ESCC cells via apoptosis and autophagy

**Figure 3:**
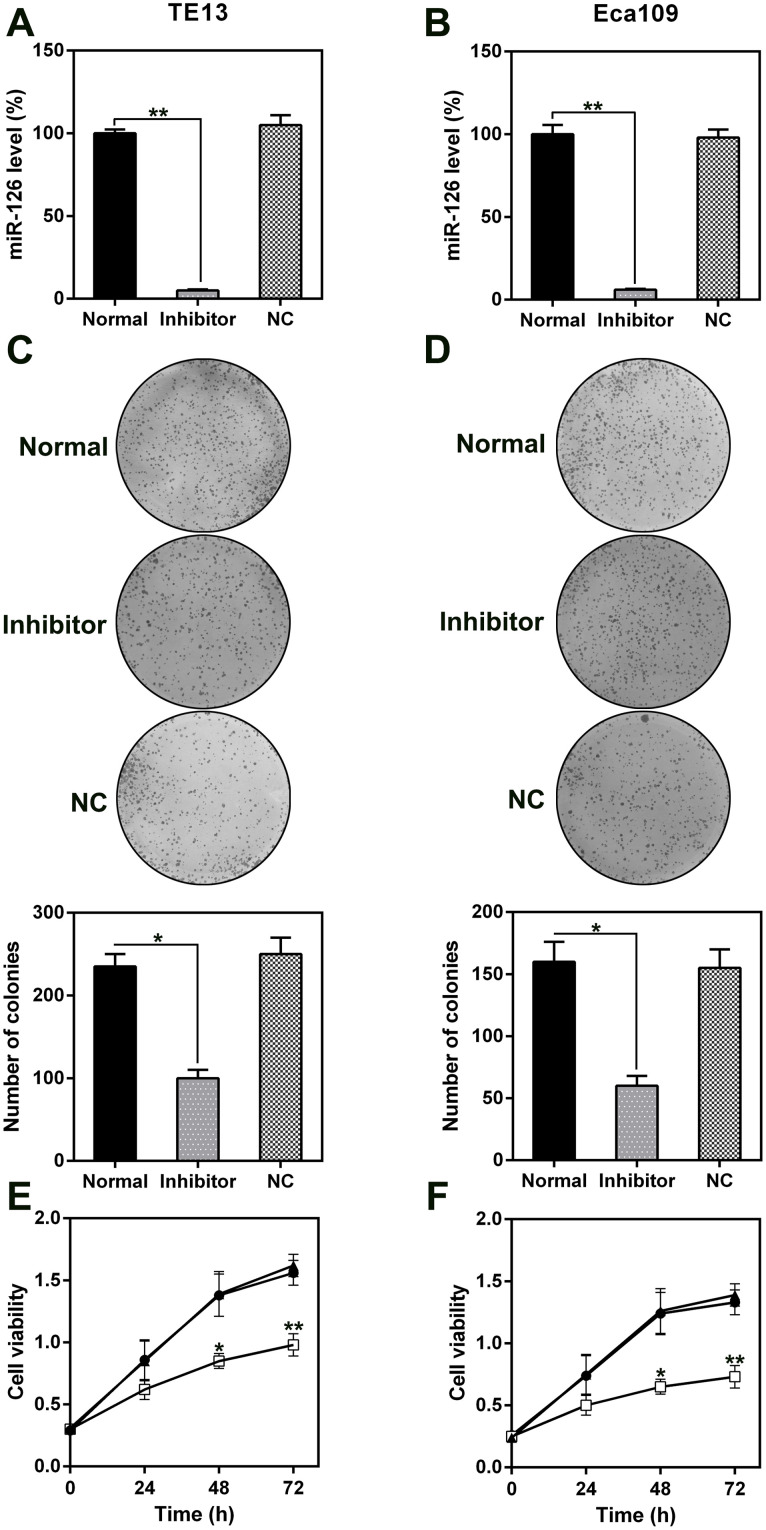
**Effect of miR-126 on ESCC cell proliferation.** (**A**, **B**) miR-126 mRNA levels in TE13 and Eca109 cells treated with miR-126 or NC inhibitors detected using qPCR. (**C**, **D**) Soft agar CFA of TE13 and Eca109 cells treated with miR-126 or inhibitors. Lower panel indicates the colony number. (**E**, **F**) TE13 and Eca109 cell proliferation rate at 1, 2, and 3 d after transfection. Results are displayed as the average ± SD. *P < 0.05, **P < 0.01, and ***P < 0.001.

The role of miR-126 in ESCC cell proliferation and viability was analyzed by transfecting TE13 and Eca109 cells with a miR-126 inhibitor. This resulted in a notable reduction in the miR-126 levels in the cells (Figure 3A, 3B). Likewise, the results of a soft agar CFA showed that aberrant miR-126 expression caused an obvious reduction in the colony numbers formed by TE13 and Eca109 cells, whereas the NC-inhibitor had no effect (Figure 3C, 3D). The results of the MTT assay indicated that TE13 and Eca109 cell proliferation was noticeably inhibited at 1, 2, and 3 d after transfection with the miR-126 inhibitor (Figure 3E, 3F).

**Figure 4:**
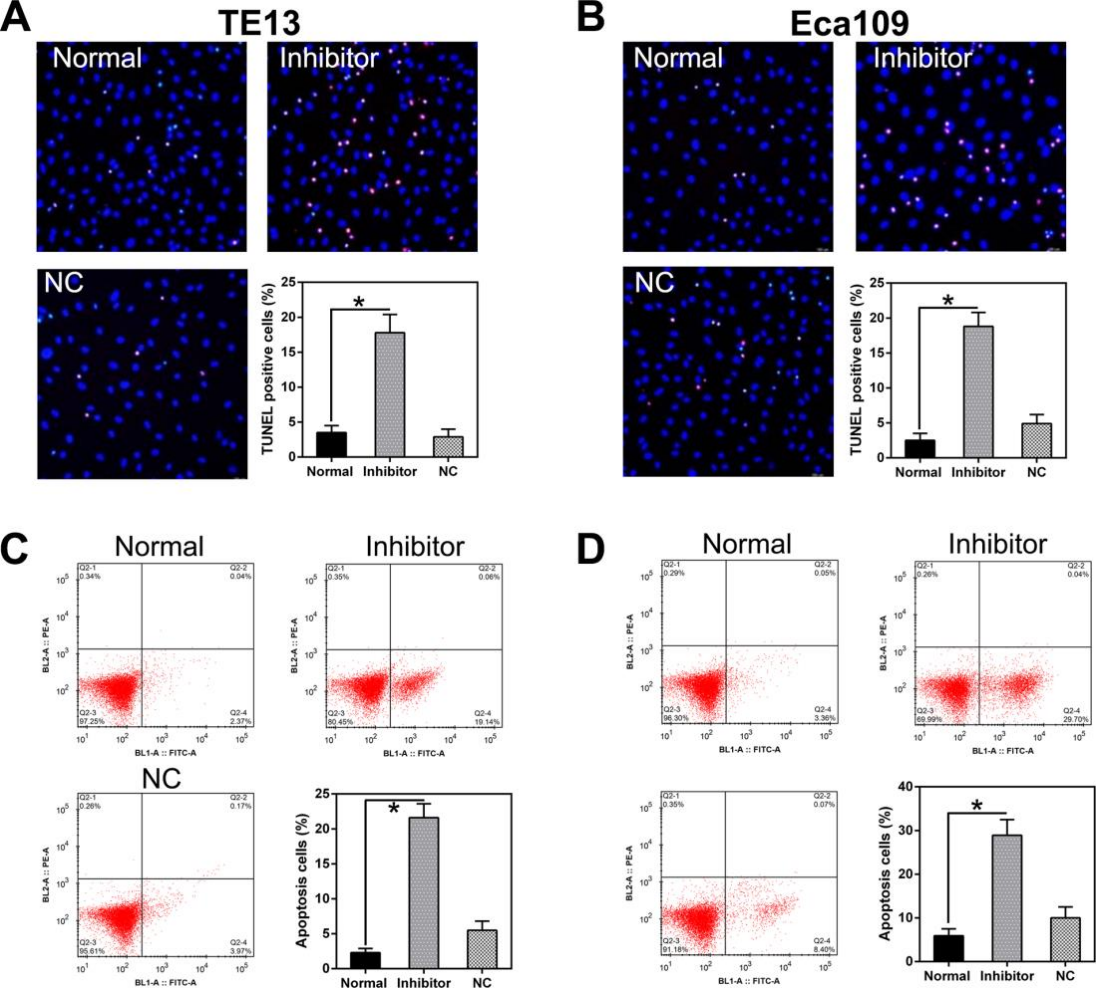
**miR-126 inhibition increases apoptosis in ESCC cells.** (**A**, **B**) TUNEL staining was used to detect apoptosis in TE13 and Eca109 cells. Magnification, × 100. Apoptotic rate is indicated in the lower right panel. (**C**, **D**) Apoptotic cell number is displayed in the first quadrant. Apoptotic rate is displayed in the lower right panel. Results are displayed as the average ± SD. *P < 0.05, **P < 0.01, and ***P < 0.001.

miR-126 silencing attenuated TE13 and Eca109 cell proliferation and viability; we speculated that this may be due to the stimulation of apoptosis or autophagy in the cells. TUNEL staining (Figure 4A, 4B) and flow cytometry (Figure 4C, 4D) did indeed confirm that a higher level of apoptosis occurred in PC cells treated with the miR-126 inhibitor than in normal cells or cells transfected with the NC-inhibitor.

**Figure 5:**
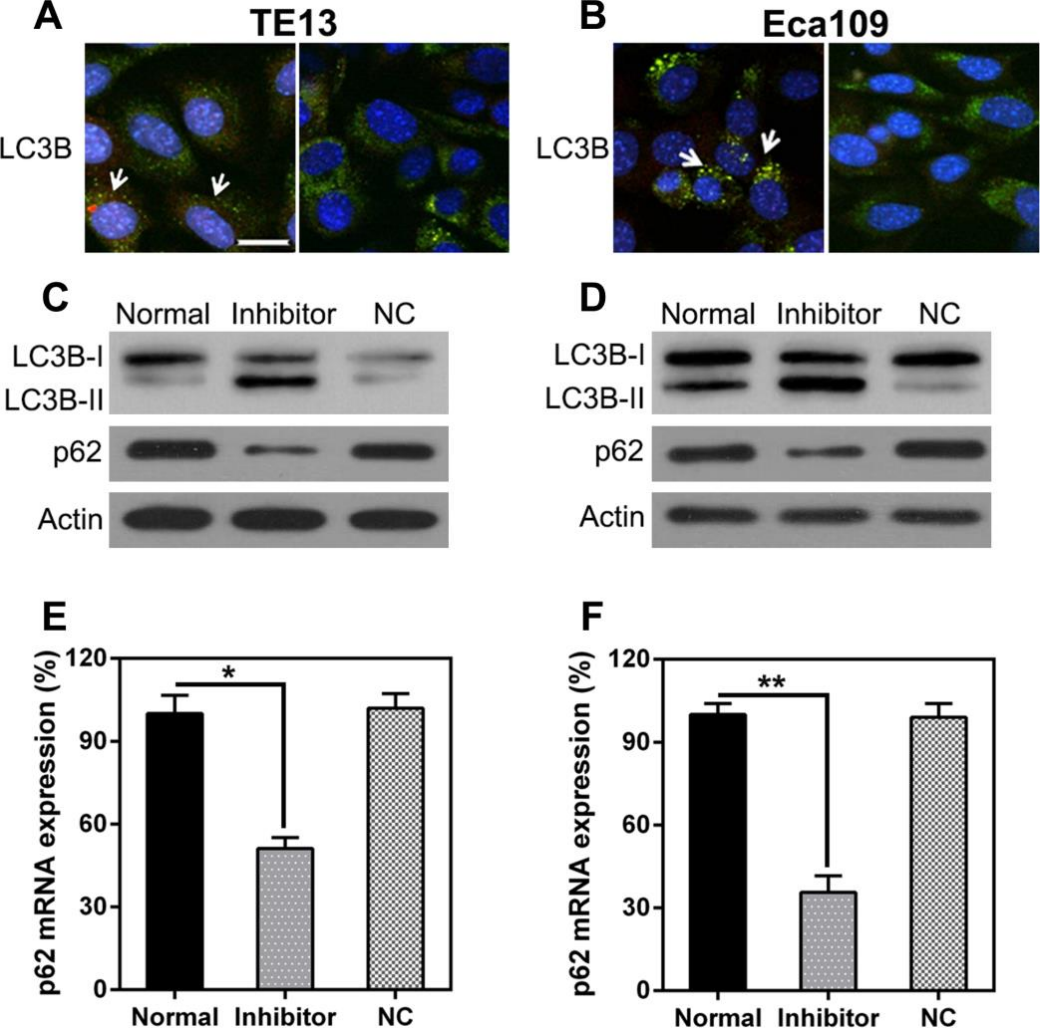
**miR-126 depletion leads to autophagy.** (**A**, **B**) TE13 and Eca109 cells were plated onto 24-well plates and treated with GFP-LC3 plus either the miR-126 or NC-inhibitor for 36 h before harvesting. GFP-LC3B was assessed using IFA (magnification, × 400). (**C**, **D**) WB analysis of LC3B and p62 protein expression in cells after miR-126 silencing. (**E**, **F**) qPCR analysis of p62 mRNA expression after miR-126 silencing in TE13 and Eca109 cells. Results are displayed as the average ± SD. *P < 0.05, **P < 0.01, and ***P < 0.001.

The effect of miR-126 inhibition on autophagy in ESCC cells was also explored. IFA was used to detect LC3B protein expression after miR-126 silencing; the results show LC3B (green) accumulation in the cells treated with the miR-126 inhibitor (Figure 5A, 5B). Autophagy was enhanced in the two ESCC cell lines following miR-126 inhibition, as indicated by the augmented LC3B biosynthesis and processing and p62 degradation (two main indicators of autophagy) (Figure 5C, 5D). Furthermore, qPCR was used to assess p62 transcription; the results revealed a positive correlation with miR-126 concentrations (Figure 5E, 5F). These results demonstrate that miR-126 may inhibit ESCC cell autophagy, which could help promote cell proliferation.

### MiR-126 targeted the 3’-UTR of JAK1

**Figure 6:**
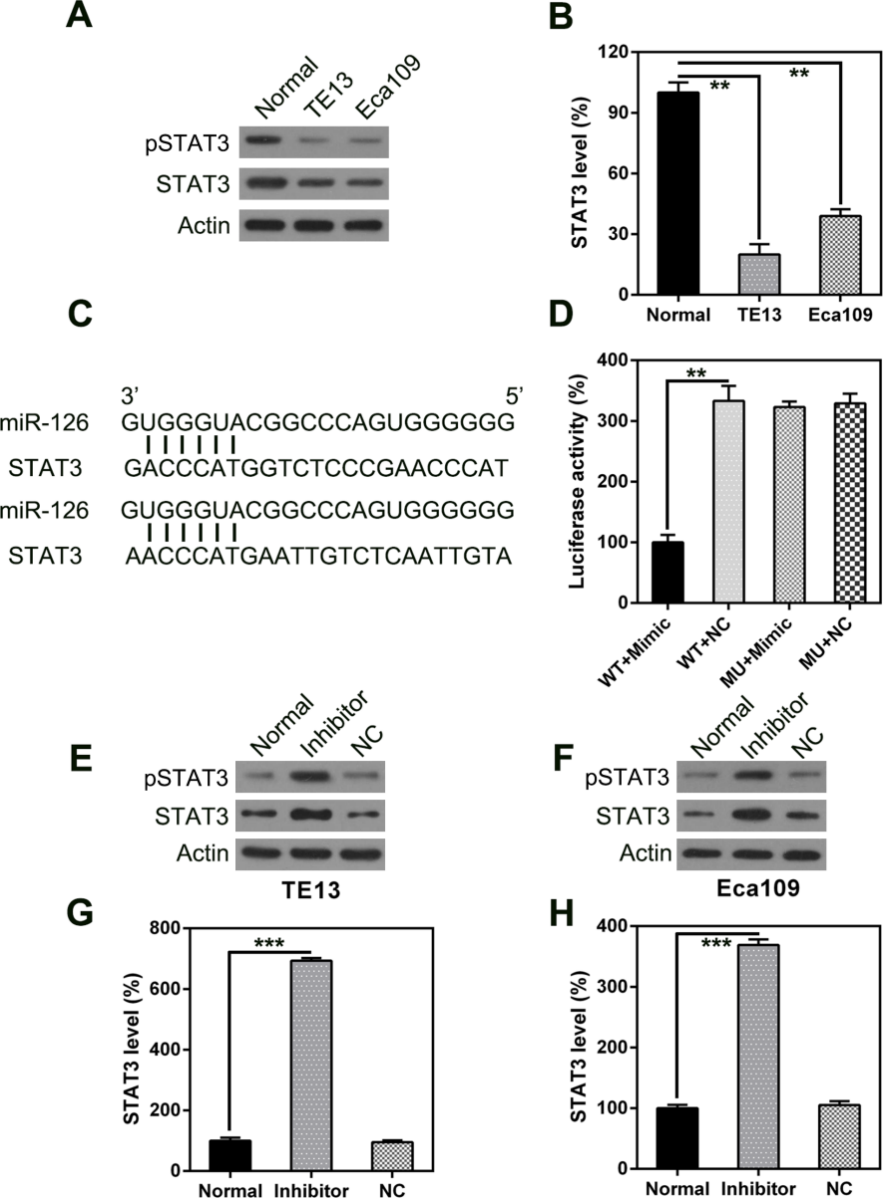
**STAT3 is a direct target of miR-126.** WB (**A**) and qPCR (**B**) were used to measure STAT3 expression in ESCC cells. (**C**) Graphical illustration of the conservative miR-126 binding motif in the STAT3 3’-UTR. (**D**) DLRA with luciferase reporter constructs of WT or MU STAT3 3’-UTR following transfection with the miR-126 mimic. Luciferase activity was standardized to β-galactosidase. Treatment with the miR-126 mimic dramatically reduced the relative luciferase activity in the WT 3’-UTR. WB (**E**, **F**) and qPCR (**G**, **H**) were used to measure STAT3 protein and mRNA expression following transfection with miR-126 or NC inhibitors. Results are displayed as the average ± SD. *P < 0.05, **P < 0.01, and ***P < 0.001.

STAT3 has been reported to be associated with apoptosis and autophagy in various cell types [17–21]. Therefore, we analyzed STAT3 expression in ESCC cells; we observed higher expression in ESCC tumors than in normal esophageal cells, at both the mRNA and protein level (Figure 6A, 6B). Furthermore, bio-informatics prediction suggests that miR-126 may target the 3’-UTR of STAT3. A possible direct link between miR-126 and the STAT3 3’-UTR was examined using a DLRA (Figure 6C). The results indicate that luciferase activity was 70% lower after transfection of the miR-126 mimic than in the control groups (Figure 6D).

The effect of miR-126 inhibition on STAT3 expression in TE13 and Eca109 cells was determined using qPCR and WB. The expression levels of STAT3 mRNA and protein were found to be upregulated following transfection of the miR-126 inhibitor (Figure 6E–6H). These findings demonstrate that STAT3 expression is increased after miR-126 silencing and suggest that miR-126 may target the STAT3 3’-UTR.

### *STAT3* knock-down attenuated the effect of miR-126 silencing on ESCC cell viability

**Figure 7:**
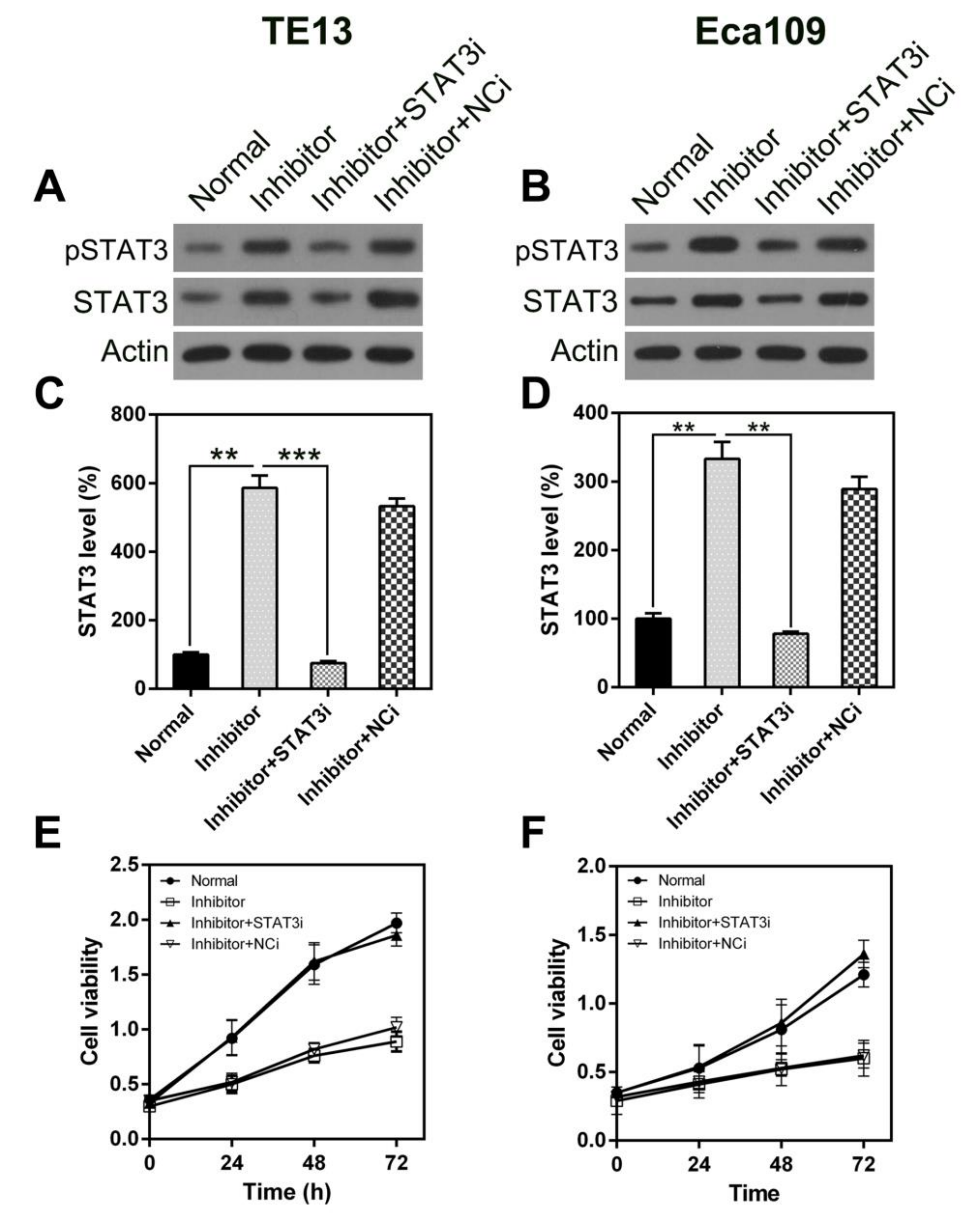
***STAT3* silencing rescues the inhibitory effect of miR-126 on ESCC cell proliferation.** STAT3 expression was detected in TE13 and Eca109 cells at both the protein (**A**, **B**) and mRNA (**C**, **D**) levels. STAT3i represents RNA knock-down of *STAT3*. (**E**, **F**) *STAT3* knock-down affected the proliferation of TE13 and Eca109 cells.

**Figure 8:**
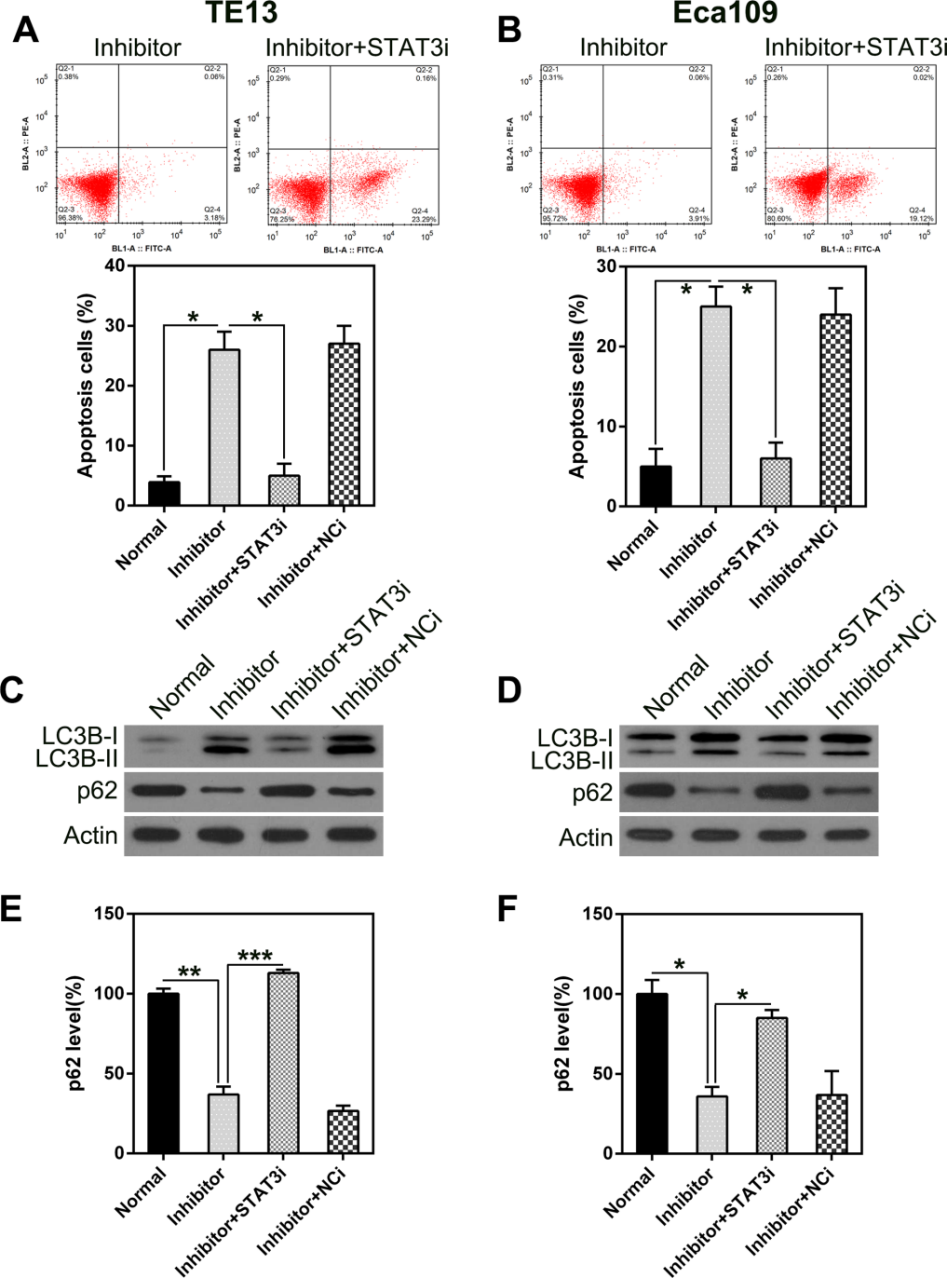
***STAT3* silencing suppresses the apoptosis and autophagy in ESCC cells caused by miR-126 silencing.** ESCC cells were treated with an miR-126 inhibitor plus shRNA-STAT3 or shRNA-NC. (**A**, **B**) *STAT3* knock-down eliminated the increase in apoptosis caused by miR-126 inhibition. FC with annexin V-FITC and PI staining was used to assess early apoptosis in TE13 and Eca109 cells at 36 h post transfection. (**C**, **D**) WB was used to examine LC3B and p62 protein levels in cells after transfection. (**E**, **F**) qPCR was used to measure p62 mRNA expression in TE13 and Eca109 cells after different treatments. Results are displayed as the average ± SD. *P < 0.05, **P < 0.01, and ***P < 0.001.

To determine whether *STAT3* knock-down inhibits the effect of miR-126 on ESCC cell viability, including apoptosis and autophagy, we silenced *STAT3* transcription in TE13 and Eca109 cells following treatment with the miR-126 inhibitor. qPCR and WB were used to assess the changes in *STAT3* expression (Figure 7A–7D). An MTT assay was also used to evaluate the effect on cell proliferation (Figure 7E, 7F). To assess the role of STAT3 in ESCC cell apoptosis, we performed a TUNEL assay on TE13 and Eca109 cells that were treated with the miR-126 inhibitor. FC results showed that aberrant STAT3 expression caused an obvious reduction in the number of apoptotic ESCC cells (Figure 8A, 8B).

In addition, *STAT3* silencing led to a decrease in autophagy, as indicated by the attenuated LC3B biosynthesis and processing and p62 stabilization (Figure 8C, 8D). qPCR was used to detect p62 transcription. The results showed an inverse correlation with STAT3 expression (Figure 8E, 8F). These findings indicate that *STAT3* knock-down may restore the reduction in proliferation caused by miR-126 inhibition-induced apoptosis and autophagy.

## DISCUSSION

Tumorigenesis is a synergistic process involving the activation of multiple oncogenes, tumor inhibitor genes, and epigenetic alterations, all of which lead to tumor formation. ESCC has been widely studied, but the underlying mechanism is still unclear. In the present study, miR-126 was found to be upregulated in two different ESCC cell lines (TE13 and Eca109). miR-126 inhibition suppressed viability and proliferation in these cells, while inducing apoptosis and autophagy. *In vivo* experiments showed that miR-126 knock-down inhibited ESCC tumor growth in mice and inhibited the expression of *STAT3*, an essential oncogene and therapeutic target. Further investigation revealed that miR-126 may directly bind to the STAT3 3’-UTR and abrogate its expression. *STAT3* inhibition may also rescue ESCC from the effects of miR-126 inhibition. These observations strongly suggest that miR-126 may suppress ESCC tumorigenesis by modulating cell viability and migration, by acting on *STAT3*.

MiRNAs are considered to influence biological processes by regulating target genes. Several studies have shown that miR-126 targets include *ADAM9* [22], LRP6, *PIK3R2* [23], *CXCR4* [24], and *SLC7A5* [25], all of which participate in the development of diverse tumor types. In the current work, we used a bio-informatics analysis to predict that miR-126 may target a conserved *STAT3* site. Previous DLRA results showed that *STAT3* acts directly on miR-126 in ESCC and plays a role in many diverse biological processes [26], whereas phosphorylated STAT3 regulates a number of different genes, including Bcl-xL, Bcl-2, VEGF, and MMPs [26–29]. Previous research has shown that the STAT3 pathway may be related to different aspects of autophagy [30]. A complex set of pathways regulates autophagy, and their interaction with other stress-response pathways, such as the STAT3 pathway, could influence cell survival or death [31]. In the present work, STAT3 was found to be augmented after miR-126 inhibition, and knock-down of *STAT3* in ESCC cell lines restored the miR-126-induced changes in the cells. These observations suggest that miR-126 may have a prominent role in ESCC tumorigenesis and may be regulated by STAT3.

In summary, this research provides strong evidence that miR-126 may have a multi-functional effect on oncogenes, as suggested by the major changes observed in the ESCC cell lines and ESCC tumorigenesis in the mouse model. Nevertheless, we are unable to exclude other factors that miR-126 may target, in addition to *STAT3*, and that contribute to ESCC pathogenesis. In future studies, an improved HITS-CLIP and MiR-Trap method, described by Baigude et al. [32], could be used to identify the targets of biotin-marked miR-126 in ESCC cells. This may provide a more exact and elaborate interpretation of the role of miR-126 in ESCC progression.

## MATERIALS AND METHODS

### Cell lines and culture

Eca109 and TE13 (ESCC cell lines) were purchased from Beijing Medical College (Beijing, China) and cultured in RPMI 1640 medium containing 10% FBS (Invitrogen, Carlsbad, CA, USA) under 5% CO_2_ at 37°C.

### Tissue specimens

Twenty patients with esophageal illness were enrolled in the Nursing Department, Affiliated Hospital of Jining Medical University (Jining, China). Patients who had undergone chemotherapy, radiotherapy, or other esophageal operations before the esophagectomy were excluded from the study. Twenty pairs of ESCC and matching healthy esophageal tissues (five centimeters from the malignant lesions according to the NCCN guidelines for EC) were acquired. Patients who received postoperative pathological diagnoses of both intra-epithelial neoplasia (IEN) and ESCC formed the ESCC group, whereas those who were diagnosed with IEN only formed the IEN group. All specimens were snap-frozen in liquid N_2_ and stored at -80°C. The study was approved by the local ethics committee, and all participants provided written informed consent.

### *In vivo* tumorigenesis experiment

Male nude BALB/c nu/nu mice aged 6 weeks or older, lacking a thymus, were used for tumorigenicity evaluation. To examine the impact of miR-126 exhaustion on malignancy generation, we transplanted TE13-miR-126i or TE13-NC cells subcutaneously into the dorsal flank of recipient mice (1 × 10^7^ cells/ml). The volume of the malignancy was assessed every 5 days. Mice were sacrificed after 30 days, and biopsies were carried out. The weight of the malignancies was estimated. Malignancy volume was determined according to the equation: V=A × B^2^/2, where A represents the maximal diameter and B represents the A-perpendicular diameter. Five mice were included in each group. All procedures complied with the institutional guidelines.

### Transfection

Hsa-miR-126 mimic, miRNA-126 inhibitor, and their corresponding negative controls (NC-mimic, NC-inhibitor) were purchased from RiboBio (Guangzhou, China). The mimics and NC-mimics consisted of incomplete complementary double strands, whereas the inhibitors and NC-inhibitors consisted of a single strand. ESCC cells were plated overnight. Transient transfections were performed using Lipofectamine 2000 (Invitrogen) according to the manufacturer’s instructions. Cells were cultured for 24 or 48 h before being analyzed.

### Western blotting (WB)

RIPA buffer (pH 8.0), including 150 mM NaCl, 0.1 % SDS, 50 mM Tris-HCl, and 1% NP-40, was added to a protease inhibitor cocktail (Roche Applied Science) and used to obtain whole cell lysates. Protein was quantified using a bicinchoninic acid protein quantitation kit and isolated via SDS-PAGE. Proteins were transferred onto a 0.450-µm PVDF membrane (Millipore, MA, USA), blocked with 5% BSA for 60 min at 25°C, and incubated with primary antibodies overnight at 4°C. Subsequently, blots were incubated with secondary antibodies for 60 min at 25°C. Immunoreactivity was determined using a Maximum Sensitivity Kit (Thermo, MA, USA). Imaging was performed using a Blot Scanner.

### RNA isolation and qPCR

Total RNA was isolated using Trizol reagent (Invitrogen CA, USA). Transcription was determined via PCR (Roche, Germany) with SYBR Green, and GADPH served as an internal reference. qPCR cycles were as follows: denaturation for 30 s at 95°C, then 30 cycles of 15 s at 95°C and 60°C for 30 s, and extension at 72°C for 30 s. The 2^-ΔΔCT^ method was used to calculate target values by standardizing to the internal reference (average of controls).

### MTT assay

Cell viability was evaluated using an MTT assay. VSMCs were supplemented with MTT (0.02 ml, 0.5 g/l) and DMSO (0.15 ml); then, the supernatant was removed, and the mixture was rotated for 10 min to incorporate the formazan dye. A_490_ was measured using an Infinite M200 microplate reader (Tecan, Männedorf, Switzerland).

### Colony formation assay (CFA)

Cells were transfected with the appropriate treatments and then resuspended in DMEM with FBS (10%) and top agar (0.4%, 8 mm) for 2 d before being moved to a 12-well plate containing bottom agar (0.5%, 0.5 ml). Two weeks later, three areas of each plate were randomly selected for colony quantification.

### TUNEL staining

To assess changes in apoptosis, we subjected the cells to a TUNEL assay (Roche, Basel, Switzerland) and counterstained them with DAPI (1:5000, Beyotime, China) to visualize the nuclei. Apoptosis was evaluated by counting the TUNEL-positive cells using an SP8 confocal microscope (Leica, Japan).

### Cell death evaluation

Cell death was evaluated using an annexin V-FITC/PI apoptosis detection kit (BD Pharmingen™). Twenty microliters of binding buffer was added to the cell suspension after transfection. Cells were incubated with 5 µl propidium iodide (PI) and 10 µl annexin V-FITC for 20 min in the dark. Flow cytometry (FC) was used to quantify the positive cells.

### Dual luciferase reporter assay (DLRA)

A 3’-UTR luciferase reporter assay was used to verify targets of miR-126 using wild type and mutant STAT3 3’-UTR. Constructs containing firefly luciferase and Renilla luciferase were prepared and used to calibrate fluorescence (Luc) and reporter fluorescence (Rluc), respectively. Cells were cultured for 24 h with vectors or miRNA mimics as appropriate. Luciferase activity was evaluated via the DLRA system.

### Immunofluorescence assay (IFA)

The human prostate cancer cell line PC-3 was treated with GFP-LC3B expression constructs (Addgene). Images were acquired using a microscope, and GFP-positive puncta from 10 distinct areas were evaluated.

### Statistical analysis

Data are expressed as the average ± SD. A two-tailed *t*-test or one-way ANOVA was applied to assess intergroup differences. P < 0.05 indicates statistical significance.
